# Highly Sensitive and Selective Graphene Nanoribbon Based Enzymatic Glucose Screen-Printed Electrochemical Sensor

**DOI:** 10.3390/s22249590

**Published:** 2022-12-07

**Authors:** Ema Gričar, Josip Radić, Boštjan Genorio, Mitja Kolar

**Affiliations:** 1 Department of Chemistry and Biochemistry, Faculty of Chemistry and Chemical Technology, University of Ljubljana, Večna pot 113, 1000 Ljubljana, Slovenia; 2 Department of Environmental Chemistry, Faculty of Chemistry and Technology, R. Boškovića 35, 21000 Split, Croatia; 3 Department of Chemical Engineering and Technical Safety, Faculty of Chemistry and Chemical Technology, University of Ljubljana, Večna pot 113, 1000 Ljubljana, Slovenia

**Keywords:** glucose oxidase, electrochemical biosensor, glucose, electrochemical analysis, graphene nanoribbons, screen-printed electrodes

## Abstract

A simple, sensitive, cost effective, and reliable enzymatic glucose biosensor was developed and tested. Nitrogen-doped heat-treated graphene oxide nanoribbons (N-htGONR) were used for modification of commercially available screen-printed carbon electrodes (SPCEs), together with MnO_2_ and glucose oxidase. The resulting sensors were optimized and used to detect glucose in a wide linear range (0.05–5.0 mM) by a simple amperometric method, where the limit of detection was determined to be 0.008 mM. (lifetime), and reproducibility studies were also carried out and yielded favorable results. The sensor was then tested against potential interfering species present in food and beverage samples before its application to real matrix. Spiked beer samples were analyzed (with glucose recovery between 93.5 and 103.5%) to demonstrate the suitability of the developed sensor towards real food and beverage sample applications.

## 1. Introduction

Glucose is a monosaccharide, present in virtually all living organisms. It is produced by plants and algae during photosynthesis, whereas in mammals, it is involved in major metabolic pathways, present in the bloodstream, and stored in the polymerous form of glycogen in liver, muscle, and other tissues [1,2]. These facts make glucose an important compound in the medical field, biology, and biochemistry. Therefore, accurate and precise determination of glucose with fast, simple, and reliable approaches is an important topic in the field of analytical chemistry. Various analytical methods were investigated and developed in order to detect glucose, such as copper iodometry, various kinds of chromatography, spectrophotometry, electrophoresis [3,4,5], and electrochemical sensing [6,7,8,9,10,11,12]. The latter is preferred because it is inexpensive, robust, and reliable. Diabetes, one of the main public health concerns in the last decades, is among the main reasons for fast developments of various glucose sensing approaches [13]. Glucose is, to this day, one of the most researched analytes in the field of electrochemical analysis, and even one of the first commercially available chemical sensors for personal use was made for glucose detection [14]. Besides the glucose sensing in the medical field, this monosaccharide is also a highly important analyte in the food industry. It is often used as a sweetener and thickener in the form of glucose syrup, and also as a substrate for yeast during fermentation [15].

Electrochemical sensors are lately becoming a more trending topic in analytical chemistry than they ever have before [16,17,18]. They are considered to be simpler and less time consuming than, for example, chromatographic techniques, and they are also robust, reliable, and suitable for use in many complex matrices, which, in some cases, completely eliminates complicated sample preparation steps [3,19]. Other properties that are favorable for electrochemical sensors include their high sensitivity, wide concentration range, low limit of detection (LOD), and, in most cases, their selectivity. Enzymatic electrochemical sensors are especially renowned for their excellent selectivity and sensitivity towards the target analyte, but they do tend to have a shorter lifespan than their non-enzymatic counterparts. The sensor, presented in this work, does use an enzyme on its surface, though, and this enzyme is glucose-1-oxidase, which was eloquently named “*an ideal enzyme*” by R. Wilson et al. [20].

There are four types of enzymes that can oxidize glucose: glucose dehydrogenases, quinoprotein glucose dehydrogenases, glucose-1-oxidases, and glucose-2-oxidases. Glucose-1-oxidase (GOX) turned out to be the most appropriate for our application because of its specificity and especially because of its high stability (lyophilized for at least two years at 0 °C and 8 years at –18 °C) [20,21]. In this work, we used GOX from *Aspergillus niger*, since its crystal structure is well known, and it was thoroughly investigated and confirmed by Hecht et al. [22]. The glucose oxidation that is facilitated by GOX (and coenzyme flavin adenine dinucleotide) in the presence of oxygen produces glucono-*δ*-lactate and a side product—hydrogen peroxide (H_2_O_2_) [14]. If the enzyme is immobilized on an electrode surface, the produced H_2_O_2_ is then eligible to undergo a further reaction. This reaction has been previously exploited in sensor fabrication [19,23,24].

Evidently, one of the current trends in electrochemical analysis is producing sensors with chemically modified (working) electrodes [17]. Taking a look at already known and used electrode materials, their main problem is low sensitivity, low selectivity, and high redox potentials towards most analytes of interest [25]. Introducing another material onto the electrode surface, chosen for either its catalytic activity, specific reaction with target analyte, overall improvement of electrochemical properties of the electrode, or other reasons, will improve the sensor’s response. This means improving sensitivity and selectivity of the sensor, as well as accelerating the kinetics of the reaction on the electrode surface [26]. Various reports suggest that electrodes modified with different nanomaterials exhibit an increased surface area, which further increases the sensitivity of the sensor due to more binding sites, as well as facilitates the immobilization of functional recognition molecules (e.g., enzymes) [27,28]. Graphene-based materials are widely used in electrochemistry because of their large surface area, biocompatibility, ease of further modification, wide potential window, and accelerated electron transport [25]. Such materials, in combination with many others, have been previously used when designing a glucose electrochemical biosensor [6,8,9,11,12,29,30]. Graphene nanoribbons are a quasi-one-dimensional carbon allotrope and, as such, have numerous advantages over graphene sheets [31,32]. They are usually produced by longitudinal unzipping of carbon nanotubes and possess, similar to their parent material, which possesses unique electronic and morphological properties, meaning an exceptionally high aspect ratio (compared to two-dimensional graphene sheets), band gap opening, and higher reactivity [33,34]. Doping such materials with electron donor elements tends to further improve the electrochemical activity, facilitate immobilization of the recognition elements, and promote charge transfer [35]. There are only a limited number of research papers covering graphene nanoribbons used in electrochemical sensors and biosensors [11,12,36,37]. However, this topic is gaining more attention in recent years.

In this work, we used a type of graphene nanoribbons that was doped with nitrogen in order to improve the sensitivity and overall electrochemical properties of the working electrode. The presented work exploits H_2_O_2_ as the side product of GOX-mediated oxidation of glucose for the indirect sensing of glucose. The combination of materials that we produced and used in the development of this sensor significantly increases its sensitivity towards glucose in comparison to similar published works. The decomposition of H_2_O_2_, produced by the above described reaction, was, in our case, facilitated with manganese dioxide (MnO_2_), since it has an exceptionally high catalytic activity towards this reaction, and is simultaneously thermally and chemically stable, environmentally friendly, as well as cost effective [38]. According to an extensive study review, the work presented in this paper is the first to successfully combine a nitrogen-doped quasi-one-dimensional graphene-based material, MnO_2_, and GOX in a simple setup in order to detect glucose.

## 2. Materials and Methods

### 2.1. Solutions

All chemicals were of analytical grade (unless stated otherwise). Phosphate buffer (0.1 M) was prepared by weighing the appropriate amounts of NaH_2_PO_4_ (Honeywell-Fluka, Charlotte, NC, USA) and Na_2_HPO_4_ (Merck, Rahway, NJ, USA) and dissolving them in ultrapure water with resistivity > 18.2 MΩ/cm (Millipore/MilliQ system; MQ). The amounts of the phosphate salts were beforehand calculated so that the resulting buffer solution would amount to the pH value of 7.4. Still, the pH value was checked and adjusted, if necessary, before the freshly prepared buffer was used in further experiments. pH value of the buffer was also checked weekly. For these measurements, we used pH meter 781 pH/Ion Meter (Metrohm, Herisau, Switzerland) and a Metrohm combined glass electrode 6.0259.100 (Metrohm, Herisau, Switzerland).

Phosphate buffer was further used as a medium for K_4_[Fe(CN)_6_]/K_3_[Fe(CN)_6_] (Merck, Rahway, NJ, USA) both solutions, as well as glucose solutions. Glucose solution was prepared from anhydrous glucose (Merck, Rahway, NJ, USA) in the form of 1 M stock solution that was further diluted, as needed. Solutions of possible interfering species (ascorbic acid (Gram-mol, Zagreb, Croatia), citric acid, sucrose, fructose, mannose, lactose, and maltose (all Sigma-Aldrich, Darmstadt, Germany)) were prepared as stock solutions with 0.1 M concentrations, respectively, and diluted as needed. GOX from *Aspergillus niger* was bought from Sigma-Aldrich, Darmstadt, Germany, and was dissolved in phosphate buffer immediately before use.

### 2.2. Synthesis and Characterization of N-htGONR

N-htGONRs were synthesized by a top-down approach from multi-walled carbon nanotubes (M-grade MWCNTs, NanoTechLabs, Yadkinville, NC, USA), according to a previously published method [39]. Other reagents used in the synthesis process were H_2_SO_4_, H_3_PO_4_, H_2_O_2_, KMnO_4_, and HCl (all obtained from Sigma-Aldrich, Darmstadt, Germany). In general, the synthesis was a two-step process i.e., the synthesis of graphene oxide nanoribbons (GONR) and heat treatment in NH_3_ atmosphere. The intermediate compound GONR was prepared by an improved Hummer’s method [40], where 20 g of MWCNTs were weighed and thoroughly oxidized with a mixture of concentrated H_2_SO_4_ and H_3_PO_4_ (vol. ratio if 9:1) and KMnO_4_. The second step included heat-treating GONR in NH_3_ atmosphere (constant gas flow of 30 mL/min) according to the following temperature program: (a) 10 K/min heating from room temperature to 800 °C, (b) hold at 800 °C for 10 min, and (c) cooling at 20 K/min from 800 °C to room temperature. The produced N-htGONRs were then characterized with the following methods: Brunauer-Emmett-Teller (BET) analysis using an ASAP 2020 Micrometrics setup (Micrometrics, Norcross, GA, USA), Raman spectroscopy with Raman/AFM WITec Alpha 300RAS (WITec, Ulm, Germany), inductively coupled plasma mass spectrometry (ICP-MS) using an ICP-MS Agilent Technologies 7900 instrument (Agilent Technologies Ltd., Santa Clara, CA, USA), C, H, and N analysis using a PerkinElmer CHN Analyzer 2400 II (PerkinElmer, Rodgau, Germany), X-ray photoelectron spectroscopy (XPS) with a PHI Quantera SXM photoelectron spectrometer analyzer (PHI, Chanhassen, MN, USA), and scanning electron microscopy (SEM) using a field emission electron microscope Zeiss ULTRA plus SEM (Carl Zeiss NTS Ltd., Oberkochen, Germany). The synthesis process and all of the mentioned characterization procedures are thoroughly described in our previously published work [39].

### 2.3. Modification of the Electrode Surface

The commercially obtained screen-printed three-electrode system DRP C-110 (Metrohm DropSens, Ovideo, Spain) was kept in the dark at room temperature and was tested by performing cyclic voltammetry (CV) in 5 mM K_4_[Fe(CN)_6_]/K_3_[Fe(CN)_6_] solutions in 0.1 M phosphate buffer before modification and further use. N-htGONR was mixed in 1:1 ratio with MnO_2_ (<1 µm particles; Sigma-Aldrich, Darmstadt, Germany) and ultrasonically dispersed in 50% ethanol, where the final concentration of each material was 1 mg/mL. To modify the working electrode, 2 µL of N-htGONR/MnO_2_ mixture was casted onto its surface, following by drying, washing, and casting 5 µL of GOX solution in 0.1 M phosphate buffer. After drying off this final layer, the electrode was thoroughly rinsed with MQ before further use. The volumes of the casted materials during the modification differ because of the different media used in both modification steps (MQ, 50% ethanol) and their respective surface tensions. The optimization of the GOX amount on the electrode surface was also carried out, where GOX solutions with concentrations between 5 and 20 mg/mL were used. When the electrodes were prepared by the above-described procedure, they were either immediately used to detect glucose, or were stored at (a) room temperature and (b) 4 °C in case of lifetime studies.

The surface morphology of thus prepared electrodes was also characterized with SEM using a field emission electron microscope, Zeiss ULTRA plus SEM (Carl Zeiss NTS Ltd., Oberkochen, Germany). The surfaces of (a) bare commercially obtained electrode, (b) electrode after the first modification step, and (c) fully prepared electrode surface were imaged. Modified SCPEs were placed on an aluminum SEM holder and secured with a conductive carbon tape. SEM images were taken at 2 kV using a SE2 detector at WD 6.1 mm.

### 2.4. Electrochemical Measurements

The electrochemical measurements were carried out on a Metrohm Autolab PGSTAT 302N (Metrohm, Herisau, Switzerland). First, the non-modified commercial SCPEs were tested for their basic electrochemical properties using a common electrochemical probe (5 mM K_4_[Fe(CN)_6_]/K_3_[Fe(CN)_6_] solution in 0.1 M phosphate buffer). Peak heights and peak-to-peak separations were observed to ensure the comparability of further results. Modified electrodes were also tested with the same probe.

Amperometric detection was employed to determine glucose concentrations. Each measurement followed the same procedure: (a) conditioning by performing 10 CV cycles in 0.1 M phosphate buffer with pH 7.4 in the potential window of −0.7 V to +0.85 V with scan rate 100 mV/s; and (b) hydrodynamic amperometric measurement at chosen operating potential. Glucose was added directly into the stirring solution in the electrochemical cell, and the current was continually recorded. The current changes after each glucose addition, indicating a linear response for the presented sensor. Finally, the sensor was tested against a real matrix, where beer samples, spiked with glucose, were injected straight into the electrochemical cell. Beer samples were degassed in an ultrasonic bath before using in any electrochemical measurements. Amperograms were again measured as follows: (1) standard glucose solution was added in the electrochemical cell; (2) beer was added in the electrochemical cell; and (3) spiked beer samples were added in the electrochemical cell. The beer was spiked so that the final concentration of glucose in the electrochemical cell after every addition would change by 0.1 mM. Three measurements were carried out, where each of the additions was replicated three times. The current changes were observed, and recovery values for glucose were calculated. Lager beer (Laško Union Brewery, Ltd., Ljubljana, Slovenia) samples were purchased from a local store.

## 3. Results and Discussion

### 3.1. Material Characteristics and Preparation of the Electrode Surface

For this work, quasi-one-dimensional graphene nanoribbon derivative N-htGONR was chosen due to its unique properties that reflect in a wide array of exciting new possibilities in the development of novel devices and materials [41]. Specifically, nitrogen doped quasi-one-dimensional nanostructures were previously successfully applied to use in a highly sensitive hydrogen peroxide sensor by our research group [42]. In the presented work, N-htGONRs were chosen because of their unique electrochemical behavior and the possibility for the precise detection of their glucose concentrations. Additionally, we observed significant improvement of electrochemical properties when N-htGONRs were ultrasonically dispersed, together with MnO_2_, in comparison to dispersing both materials separately and mixing them before casting onto the electrode surface. Wu et al. [43] reported a synergistic effect that a MnO_2_/rGONR nanocomposite has towards overall electrochemical properties and hydrogen peroxide sensing, and we observed a similar phenomenon for a joint dispersion.

Synthesized material was characterized by SEM, BET, and C, H, and N analyses, as well as ICP-MS, XPS, and Raman spectroscopy. Surface area, calculated via BET analysis, was 85.4 m^2^/g, while the content of nitrogen was 7.2 at. %. XPS analysis, which was carried out to investigate the distribution of nitrogen configurations, shows that pyridinic-N, pyrrolic-N, graphitic-N, and oxidized-N configurations were present. After deconvolution of N*1s* XPS core-level spectra, it was discovered that 44.2 at.% and 19.7 at.% of all nitrogen in the material was from pyridinic-N and pyrrolic-N, respectively. Pyridinic-N functional groups are considered to be highly reactive and one of the main reasons for improved electrocatalytic activity [44]. Therefore, this was in accordance with our requirements that precisely those functional groups were most commonly present in our material. Another aspect that contributes to the reactivity of the material is the concentration of structural defects, therefore, Raman spectra were recorded and examined. Observing the *I_D_/I_G_* ratio revealed a value of 1.28, which is an indicator of high defect density. The whole characterization procedure and detailed results were reported in our previous works [39,42]. Considering all the listed facts, it is expected that, when N-htGONR is casted onto a commercially obtained SPCE, it will significantly increase specific surface area, improve the conductivity, exhibit high reactivity, and enhance charge transfer characteristics due to the presence of N-functionalities.

Before each of the commercial SPCEs was used in sensor fabrication, its basic electrochemical parameters were checked by performing cyclic voltammetry in 5 mM K_4_[Fe(CN)_6_]/K_3_[Fe(CN)_6_] solution in 0.1 M phosphate buffer. Electrodes, whose parameters (peak currents, peak-to-peak separation) deviated more than 10% from the average, were discarded. Other electrodes were used in the manufacture of the glucose sensor. Before the actual sensor preparation, the cyclic voltammetry was employed again in order to examine the overall electrochemical properties of modified electrodes. Voltammograms of bare SPCE, SPCE/N-htGONR, SPCE/N-htGONR/MnO_2_, and SPCE/N-htGONR/MnO_2_/GOX were compared and are shown in Figure S1. It is clearly seen from the shape, position, and heights of the current peaks that introduction of N-htGONR into the system shifts the properties of the working electrode towards ideal parameters. Additionally, the response of the same four electrodes was tested towards 1.0 mM glucose solution in 0.1 M phosphate buffer, and the cyclic voltammograms are shown in Figure S2. A comparison of SPCE/N-htGONR/MnO_2_/GOX response to blank solution (0.1 M phosphate buffer) vs. 1.0 mM of glucose solution is also shown in Figure S3. In this case, one can note the appearance of a relatively well-defined current peak when GOX is introduced into the system. Bare SPCE, SPCE/N-htGONR, and SPCE/N-htGONR/MnO_2_ exhibit poorly defined peaks for glucose, or no peak at all.

**Figure 1:**
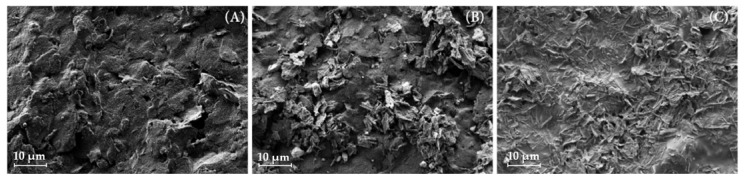
SEM images for (**A**) bare SPCE, (**B**) SPCE/N-htGONR/MnO_2_, and (**C**) SPCE/N-htGONR/MnO_2_/GOX are shown.

The final modification that was used for all described electrodes from this point on was carried out in two steps: (a) casting N-htGONR/MnO_2_ dispersion onto the electrode surface and (b) casting GOX solution onto the electrode surface. At the beginning of the research work, we decided to test a common claim that graphene-based materials aid the immobilization of various macromolecules on the electrode, especially if doped [45,46]. To test this hypothesis, 2.5 µL of N-htGONR/MnO_2_ dispersion and 5 µL of 5 mg/mL GOX solution were casted onto electrodes, their surfaces were rinsed with MQ after each modification step, and their properties and response to glucose were tested. Since the response was stable and the electrodes were usable for more than one consecutive measurement, Nafion or another similar polymer was not used in the preparation of this sensor. The surfaces of thus prepared electrodes were observed with SEM in order to obtain the insight into the morphology. Images for bare SPCE, SPCE/N-htGONR/MnO_2_, and SPCE/N-htGONR/MnO_2_/GOX are shown in Figure 1. The difference between bare (Figure 1A) and modified (Figure 1B,C) electrode surfaces morphology is obvious. Since the surface of the bare electrode is relatively rough, a further increase in specific surface area was achieved when the nanomaterials were introduced onto the electrode surface. Figure 1B shows different phases. The predominate phase belongs to quasi-one-dimensional ribbon-like flaky structures of N-htGONR, while spherical shaped particle phases belong to MnO_2_. Figure 1C shows modified electrode surface-SPCE/N-htGONR/MnO_2_/GOX (c(GOX) = 10 mg/mL). It was seen that there is an additional phase on the electrode when compared to Figure 1B. This phase was assigned to GOX. The separate GOX molecules cannot be seen using the SEM technique, since the dimensions of the globular protein are about 7 nm [47].

### 3.2. Optimization of the Modification and Operating Potential

Electrodes, modified with N-htGONR, MnO_2_, and GOX, were then tested for their response to the actual target analyte, glucose. A straightforward, robust, and reliable amperometric electroanalytical technique was employed. The setup was simple and miniaturized compared to traditional three-electrode systems, where tens of milliliters of solutions are consumed for each measurement. In our case, the medium and reagent consumption was much lower, only 5.0 mL per measurement. The modified screen-printed electrode was immersed into the medium in the electrochemical cell (0.1 M phosphate buffer with pH 7.4), the chosen operating potential was applied to the cell, and known glucose concentrations were added into the system every 100 s at constant stirring. To ensure that the working conditions and sensor response were optimal for the chosen setup, the optimization of the sensor parameters took place in the next step.

First, we deduced that it is better for the system if N-htGONR/MnO_2_ mixed dispersion and GOX solution are introduced separately. N-htGONR/MnO_2_ is dispersed in 50% ethanol because the resulting dispersion becomes homogeneous after sonicating for only 2 h (4–5 h in case of 100% MQ), and, moreover, the surface tension of such dispersion is considerably lower, meaning we could depose thinner layers. GOX was dissolved in 0.1 M phosphate buffer with pH 7.4, and, since it was necessary to ensure it is in its native form, we could not use the same medium as in the case of N-htGONR/MnO_2_. Phosphate buffer, chosen as medium for GOX solutions, as well as for the medium in the electrochemical cell, was adjusted so that the pH was set to 7.4 for all measurements. The real matrix we chose to examine in this work, lager beer, has a slightly acidic pH (4.5). However, since it was diluted in 0.1 M phosphate buffer during the measuring process, it did not influence the pH value in the electrochemical cell.

**Figure 2:**
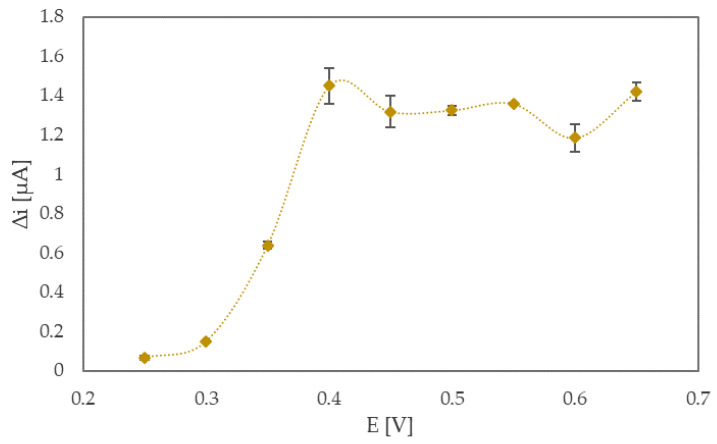
Sensor response towards 0.2 mM glucose in 0.1 M phosphate buffer (pH 7.4) measured at different operating potentials. The line was smoothed with Microsoft Excel.

The working potential was optimized in the next step by the following procedure: a N-htGONR/MnO_2_/GOX (5 mg/mL) sensor was immersed in 5.0 mL of phosphate buffer (pH 7.4); and a chosen voltage was applied to the electrochemical cell, and 0.2 mM glucose aliquots were added into the cell every 100 s at constant stirring. The potential range, in which the sensor response was evaluated, was chosen according to cyclic voltammograms, shown in Figure S2. Nine measurements were carried out at different potentials (0.25, 0.30, 0.35, 0.40, 0.45, 0.50, 0.55, 0.60, 0.65 V), and the responses of glucose sensors at different potentials are shown in Figure 2. For each point in Figure 2, there were five repetitions of measurements. Two important parameters needed to be considered when choosing the optimal operating potential: first and most obvious is the current change, i.e., the sensor’s response when repeatedly adding the same amount of glucose, and the second one is that of the potential value itself. If the operating potential is too high, the electrode integrity is risked, and a number of possible interferences dramatically increases. As seen from Figure 2, the sensor has similar responses (1.45 and 1.42 µA) at 0.4 and 0.65 V. In the end, considering all above mentioned facts, 0.4 V was chosen as the optimal operating potential.

**Figure 3:**
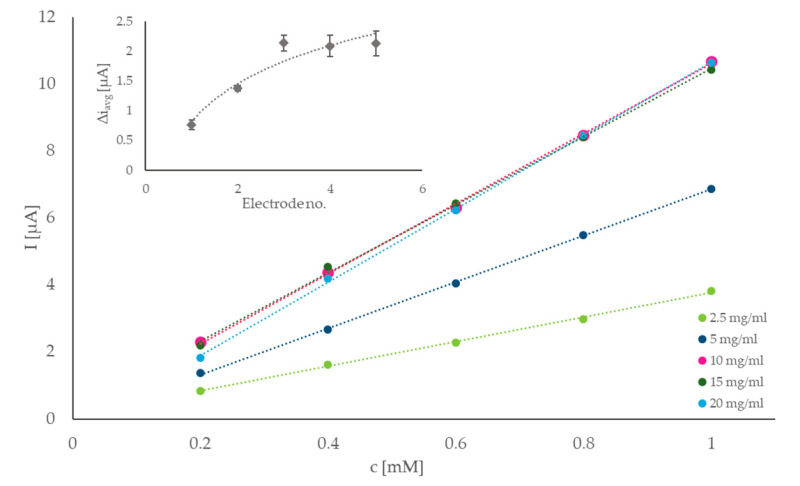
Optimization of GOX concentration on the electrode surface—the current response of electrodes with 2.5, 5.0, 10, 15, and 20 mg/mL GOX plotted against glucose concentration. Trendline slopes were used to determine the sensitivity of the electrodes. Insert: average current response of the same electrodes to repeated 0.2 mM additions of glucose.

To further optimize the response of the sensor and ensure that the maximum sensitivity was achieved with the proposed setup, the GOX concentration on the electrode surface was evaluated. Solutions with different concentrations of GOX were prepared, and 5 µL of each were cast onto the electrodes, already modified with N-htGONR/MnO_2_ composite. Five different concentrations were tested: 2.5, 5.0, 10, 15, and 20 mg/mL GOX. The sensitivities of electrodes with different GOX amounts on their surfaces were evaluated by measuring the response of the electrodes to five consecutive additions of 0.2 mM glucose solution, respectively. Current responses were calculated from the amperograms and plotted as function of glucose concentration, as seen in Figure 3. The sensitivity was determined as the trendline slope. The insert of Figure 3 shows average current response for the same set of measurements, per electrode. Both graphs in Figure 3 suggest that the current response was significantly increased with GOX concentration for the electrodes prepared with 2.5, 5.0, and 10 mg/mL GOX, and that there is not much difference between the responses of electrodes with 10, 15, and 20 mg/mL GOX on their surface. Since every electrode is thoroughly rinsed with MQ after its modification and before use, a simple deduction suggests that around 10 mg/mL GOX, which represents most of the possible immobilization sites on the electrode, are already occupied, and the increase in the concentration of the enzyme will not contribute significantly to the sensitivity of the sensor. Additionally, the enzymes are generally expensive, therefore lower consumption of GOX is desired. Considering all the data and reagent consumption, 10 mg/mL was chosen as the optimal concentration of GOX.

### 3.3. Sensor Properties

**Figure 4:**
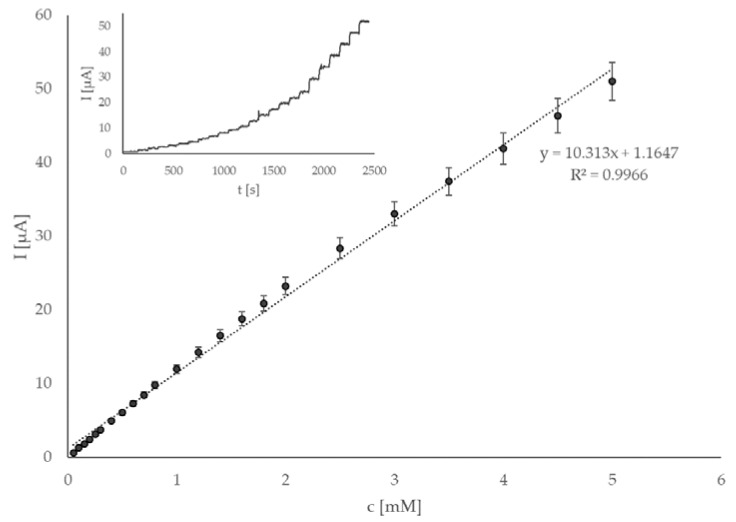
Calibration curve obtained by adding six times 0.05 mM, five times 0.1 mM, six times 0.2 mM, and six times 0.5 mM aliquots of glucose standard solutions prepared in 0.1 M phosphate buffer solution, with pH 7.4, at operating potential 0.4 V. The insert shows the amperogram of the same measurement.

General sensor parameters, i.e., linear range, LOD, sensitivity, lifetime, and reproducibility, were investigated. If the sensor is to be applied in real matrices, it has to be ensured that its response is linear and well defined in a wide and appropriate concentration range. The amperometric response for known amounts of glucose in a continuously stirred 0.1 M phosphate buffer with pH 7.4 was investigated at 0.4 V. Successive additions of glucose were pipetted straight into the electrochemical cell. The calibration curve obtained this way shows a wide linear range of 0.05 to 5.0 mM glucose. Considering the blood levels of glucose and its concentration in various food and beverage samples (such as juices and soft drinks), this concentration range is suitable for measuring such concentrations. A representative example of thus obtained amperogram and calibration curve is shown in Figure 4. The sensitivity of the proposed sensor is 10.31 µA mM^–1^ or, considering the geometric surface of the SPCE (0.126 cm^2^), 82.07 µA mM^–1^ cm^–2^, which is a significant improvement compared to most of previously published works on similar sensor systems. LOD was calculated according to the 3 s/k method [48], using 0.05 mM additions, and was found to be 0.008 mM, which was extremely satisfactory considering the simultaneous high sensitivity of the sensor. The comparison with other state-of-the-art enzymatic and non-enzymatic sensors is shown in Table 1. When comparing the parameters in Table 1, it is clearly seen that the sensor presented in this work has the lowest LOD of the selected works. Similarly, its sensitivity was significantly improved compared to other recently reported sensors. Linear range is also comparable to other works. The mentioned parameters (especially the low LOD and high sensitivity) suggest that this sensor is able to sense glucose with high sensitivity and is, as such, a step in the right direction when attempting to develop a glucose sensor for real food industry samples.

**Figure 5:**
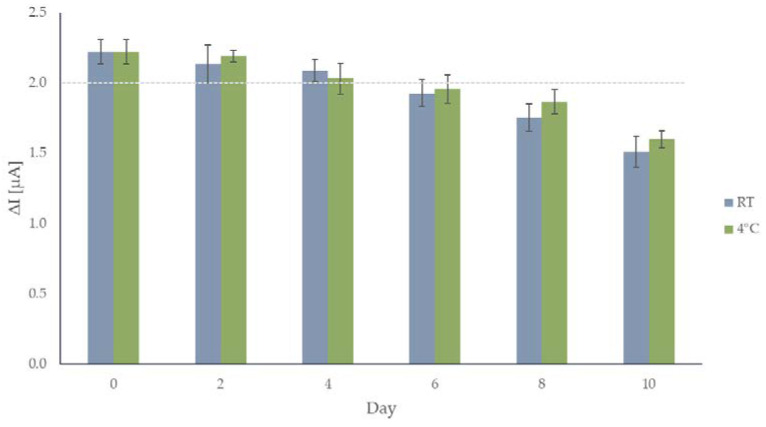
Average current responses to 0.2 mM glucose for the sensor after 0, 2, 4, 6, 8, and 10 days for the electrodes kept at room temperature and in the refrigerator. The dashed line is set at 90% of sensor’s initial response (1.998 µA).

Other important aspects that need to be considered in sensor fabrication are its lifetime and the reproducibility of the electrode surface preparation. The lifetime was studied on two sets consisting of six electrodes: the first set was kept in the dark at room temperature (RT), the second was kept in the refrigerator at 4 °C. Average responses of the sensors kept at room temperature, and in the refrigerator after 2, 4, 6, 8, and 10 days since their preparation, are shown in Figure 5, respectively. Over 90% of the response (note the dashed line in Figure 5) is definitely retained after four days in case of both sets of the electrodes, and considering the error bars, both sets of electrodes can be used for up to six days. Amperograms for both sets of electrodes after day 10 are shown in Figure S4. Since this is an enzymatic sensor, these results are acceptable and confirm the fact that GOX is a suitable enzyme for this application.

**Figure 6:**
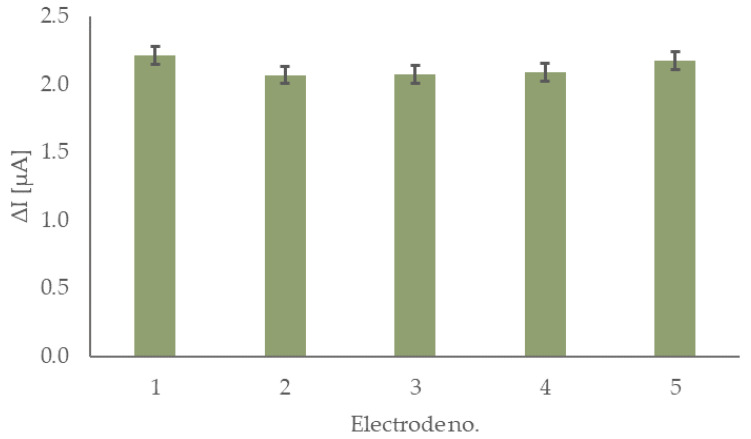
Responses of the sensor for five individually prepared electrodes.

Similar to lifetime studies, the reproducibility of the sensor preparation was also investigated, and the results are shown in Figure 6. Five electrodes, prepared on different days using the same modification approach, were tested. Their response to 0.2 mM glucose in 0.1 M phosphate buffer (pH 7.4) was measured. Five consecutive additions of 0.2 mM glucose were performed for each electrode. The calculated relative standard deviation (RSD) value for the response is 3.3%, which confirms the robustness of this sensor fabrication approach. Considering all the collected data presented in this chapter, it was decided that the sensor is ready to be tested against common interferences and in a real matrix.

### 3.4. Interference Studies and Measurements in Real Matrix

The presented sensor was tested against commonly present interfering species in food and beverage samples before its application to a real matrix. Ascorbic acid, citric acid, sucrose, maltose, fructose, lactose, mannose, and galactose were tested. Ascorbic acid has its redox potential in neutral pH region at –0.079 V [50], which means that it could strongly influence glucose measurements at our operating potential. Citric acid has its redox potential at +1.1 V [51] and was not expected to interfere with the glucose measurements. It was included in the list of tested possible interferences because it is a commonly used acidity regulator in processed food samples. Other monosaccharides and disaccharides were tested because of their common presence in processed food samples and to confirm the high specificity of GOX.

To test the sensor’s response against mentioned possible interferences, amperometric measurements were carried out. The same concentrations of glucose and possible interfering species were added into the electrochemical cell, and current response was measured. When investigating ascorbic acid, the addition of the same concertation (0.2 mM) as glucose caused a big spike of current (>2 µA), the amperogram became very noisy, and it was difficult to measure even 0.5 mM of glucose after this occurrence. We concluded that high concentrations of ascorbic acid should be avoided or removed from the sample, if possible, when using the proposed sensor. However, when ascorbic acid concentration is considerably lower than that of glucose, it does not influence the sensor’s response significantly. A sample of an amperogram from interference studies is attached in Figure S5, and the visual representation of the sensor’s response to investigated interferences is shown in Figure S6. Ascorbic acid was added in 1:10 and 1:100 ascorbic acid: glucose ratios and did not interfere with the current response. In fact, all possible interfering species change the sensor’s response for less than 5% when added in the electrochemical cell in the same concentration as glucose. This confirms the excellent specificity of GOX and high sensitivity towards glucose.

Finally, the sensor was used to detect glucose in spiked beer samples to demonstrate its applicability in real sample analysis. Beer samples were degassed, spiked with glucose, and added straight into the electrochemical cell during amperometric measurements, as described in Section 2.4. By separately adding standard glucose solution, beer, and spiked beer, we made sure that the matrix effect was either evaluated or absent. Before the final amperometric measurements, it was vital to verify that the pH of the phosphate buffer would not change significantly when adding the beer matrix into the electrochemical cell. Since lager beer, used in this measurement, has its pH value between 4 and 5, it was predicted that small additions into the electrochemical cell would not influence the pH value significantly. This was confirmed by continuously measuring the pH of 5.0 mL phosphate buffer while adding 50 µL aliquots of degassed beer sample into the buffer solution. The pH value remained at 7.4 after at least six consecutive additions of beer, which was adequate for the planned real matrix measurements.

The amperometric measurement was replicated three times, and each measurement included three additions of glucose, beer, and spiked beer, respectively. An amperogram of such measurement is shown in Figure S7. Glucose recovery was calculated for spiked beer samples from current responses for each addition. The recovery values were 96.0%, 93.5%, and 103.5% for each measurement, respectively. Considering those recovery values, the beer matrix does not significantly affect glucose measurements with the presented sensor. These results suggest that the developed sensor is appropriate to use in further food and beverage sample analysis.

## 4. Conclusions

The presented work introduces an enzymatic glucose sensor based on quasi-one-dimensional graphene nanoribbon-based N-htGONR, MnO_2_, and GOX composite. The setup was miniaturized in comparison to traditional three-electrode systems, and measuring procedures were simplified as much as possible. Glucose was determined using an amperometric approach, where operating potential and whole electrode modification procedure were optimized. The optimal working potential, used in further measurements, was 0.40 V. Linear range of the sensor was tested and was between 0.05 and 5 mM, the sensitivity was 10.313 µA/mM, while the calculated LOD was 0.008 mM. The sensor was tested for the reproducibility of its preparation, where the responses of five different electrodes were tested and compared. The RSD of such measurements was 3.3%. Lifetime of this enzymatic sensor was also investigated, and it was concluded that the sensor retains over 90% of its initial response for four days, regardless of the storage temperature (25 °C or 4 °C). We then considered common interferences and their possible influence on the response of the sensor when analyzing real food and beverage samples. Ascorbic acid, citric acid, sucrose, maltose, fructose, lactose, mannose, and galactose were tested, and it was concluded that they do not interfere with the response when investigating the expected ratios actually present in target real samples. Lastly, the sensor was used to analyze spiked beer samples, and the recovery values for glucose were between 93.5 and 103.5%.

## Figures and Tables

**Table 1:** Comparison of the main sensor parameters for previously reported glucose sensors and our work.

Linear Range [mM]	Sensitivity	LOD [mM]	Ref.
0.056–5.55	2.19 µA mM^−1^	0.0183	[49]
0.04–16.1	0.25 µA mM^−1^ cm^−2^	0.012	[10]
0.5–1.3	56.32 µA mM^−1^ cm^−2^	0.22	[11]
0.05–5.0	82.05 µA mM^−1^ cm^−2^	0.008	this work

## Data Availability

Data sharing not applicable.

## Supplementary Materials

The following supporting information can be downloaded at: https://www.mdpi.com/article/10.3390/s22249590/s1, Figure S1: Observation of general electrochemical properties.; Figure S2: Observation of electrodes’ response to glucose.; Figure S3: Cyclic voltammetry of 1.0 mM glucose in comparison to blank solution.; Figure S4: Current response of the sensor after 10 days of storage at room temperature or in the refrigerator.; Figure S5: Amperogram for interference studies.; Figure S6: A visual representation of sensor current responses for chosen interferences.; Figure S7: Amperogram for one of the real matrix measurements.
