# Supplementary material for: Highly Sensitive and Selective Graphene Nanoribbon Based Enzymatic Glucose Screen-Printed Electrochemical Sensor
Source: Sensors (Basel). 2022 Dec 7;22(24):9590. doi: 10.3390/s22249590 (PMC9786066; doi:10.3390/s22249590)
Supplement: Supplementary file 1 [file sensors-22-09590-s001.zip › sensors-2067364-supplementary.pdf]

Supplementary materials

Highly Sensitive and Selective Graphene Nanoribbon Based Enzymatic Glucose Screen-Printed Electrochemical Sensor

Ema Gričar [1], Josip Radić [2], Boštjan Genorio [3,*] and Mitja Kolar [1,*]

[1] Department of Chemistry and Biochemistry, Faculty of Chemistry and Chemical Technology, University of Ljubljana, Večna pot 113, 1000 Ljubljana, Slovenia
[2] Department of Environmental Chemistry, Faculty of Chemistry and Technology, R. Boškovića 35, 21000 Split, Croatia
[3] Department of Chemical Engineering and Technical Safety, Faculty of Chemistry and Chemical Technology, University of Ljubljana, Večna pot 113, 1000 Ljubljana, Slovenia
* Correspondence: bostjan.genorio@fkkt.uni-lj.si (B.G.); mitja.kolar@fkkt.uni-lj.si (M.K.)

Table of contents

Figure S1 …………………………………………………………………….…… 3

Figure S2. ……………………………………..……………………….……... 3

Figure S3. ……………………………………..……………………….……... 4

Figure S4 …………………………………………………………………….…… 4

Figure S5. ……………………………………..……………………….…….... 5

Figure S6. ……………………………………..………………….……..…….. 6

Figure S7………………………………………………………………………... 7

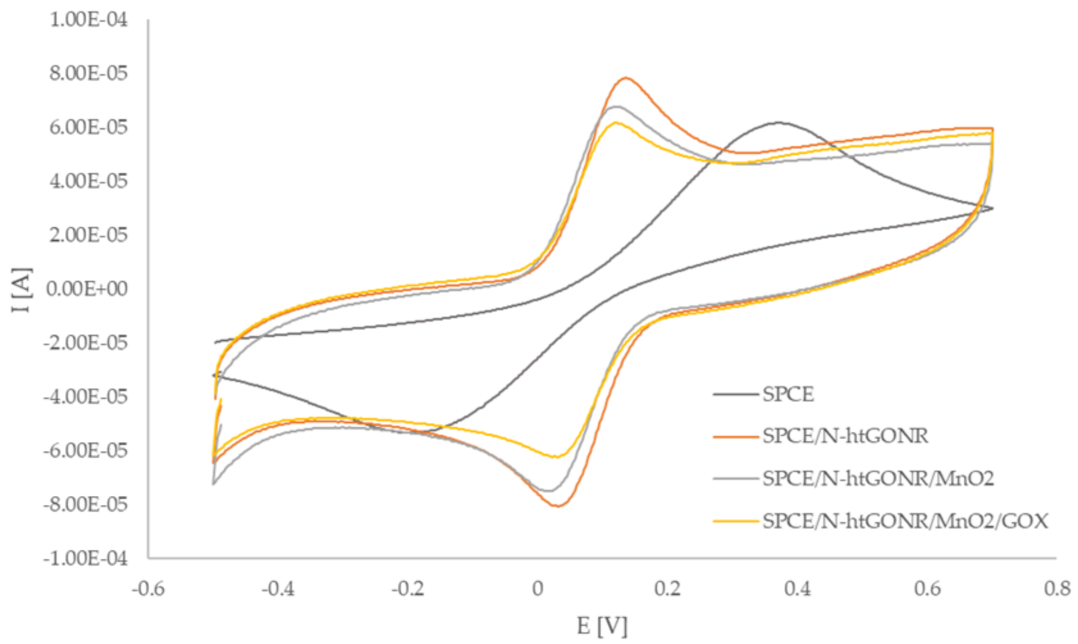

Figure S1. Cyclic voltammograms for bare SPCE, SPCE/N-htGONR, SPCE/N-htGONR/MnO_2, and SPCE/N-htGONR/MnO_2/GOX in 5 mM $K_4[Fe(CN)_6]/K_3[Fe(CN)_6]$ solution in 0.1 M phosphate buffer. Peak-to-peak separations and peak current heights suggest improvement of general electrochemical properties of the electrode when N-htGONR is introduces into the system.

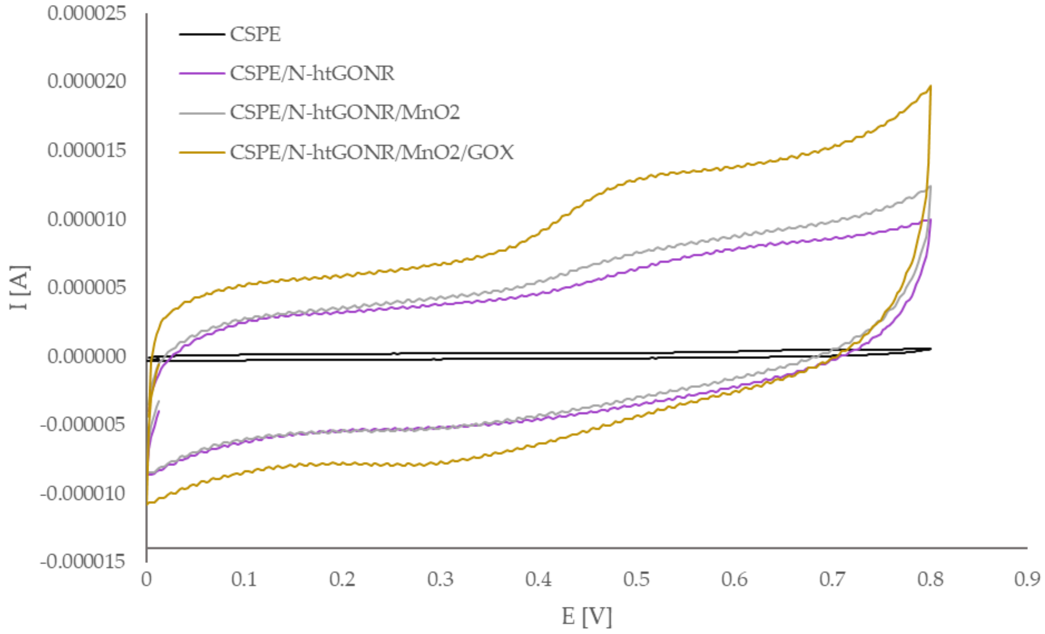

Figure S2: Cyclic voltammograms for bare SPCE, SPCE/N-htGONR, SPCE/N-htGONR/MnO_2, and SPCE/N-htGONR/MnO_2/GOX in 1.0 mM glucose solution in 0.1 M phosphate buffer (pH 7.4).

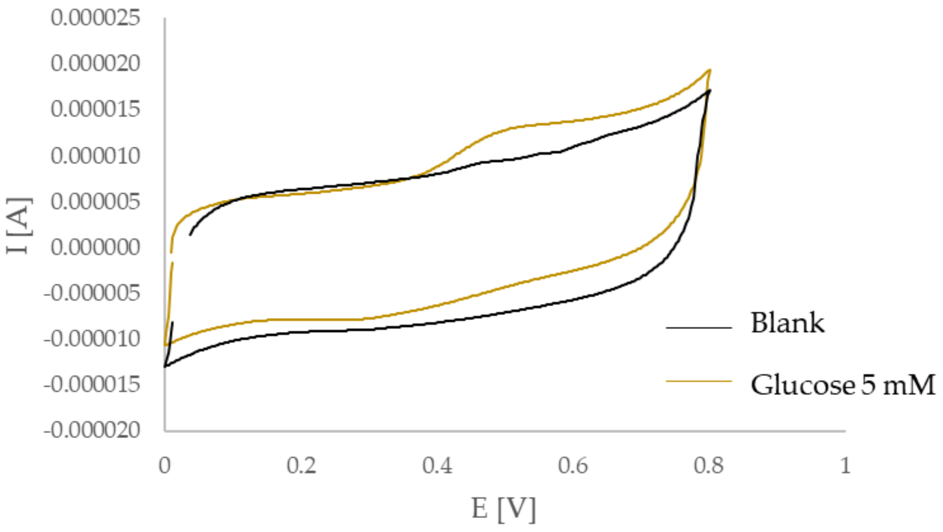

Figure S3: Cyclic voltammetry of 1 mM glucose in comparison to blank solution (0.1 M phosphate buffer). The working electrode was the same SPCE/N-htGONR/MnO_2/GOX in both cases.

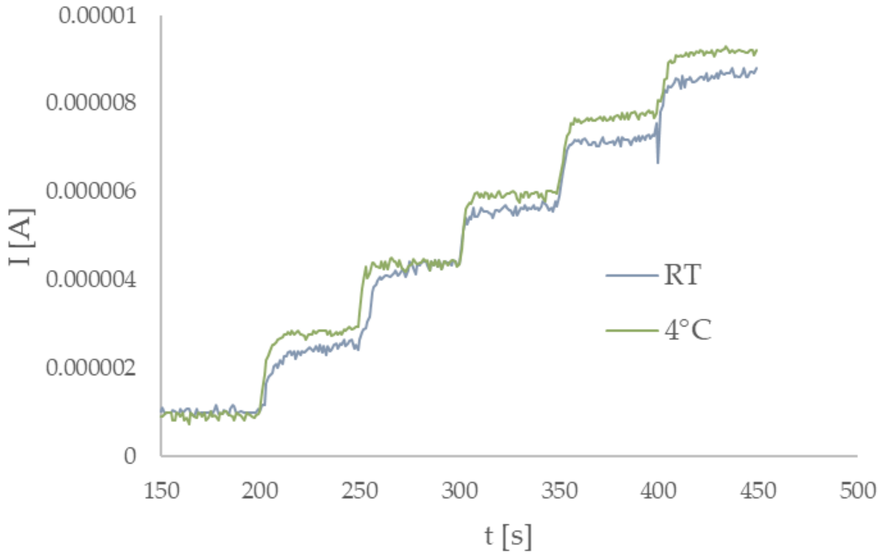

Figure S4: Current response for the presented glucose sensor after 10 days of storage at either room temperature (blue line) or refrigerated at 4 °C (green line). The average sensor response for

is 1.51 μA, and 1.60 μA for room-temperature-stored and refrigerated sensors, respectively.

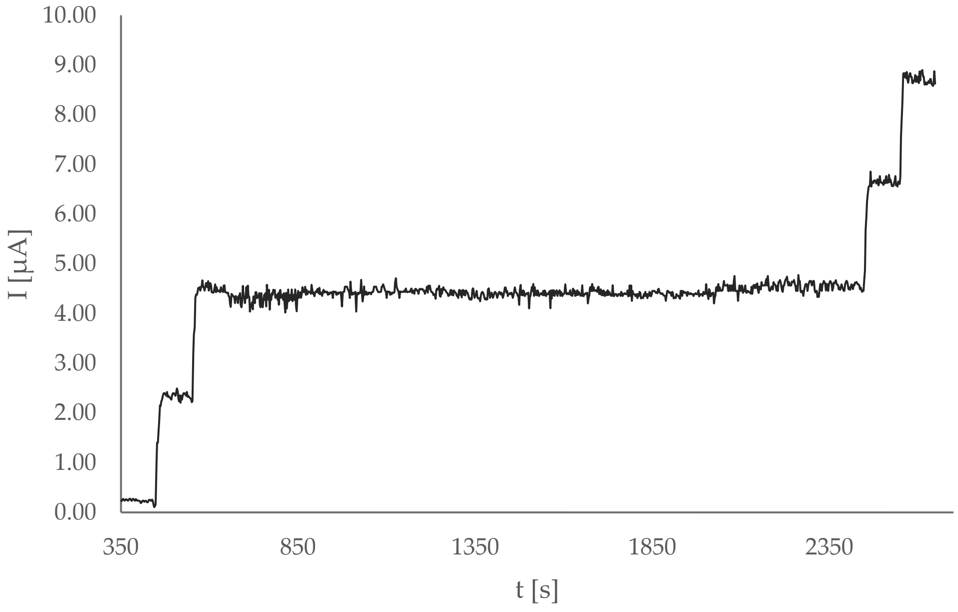

Figure S5: Amperogram for interference studies. Glucose was added twice before the addition of each interference (twice), and twice after the addition of all interferences. Glucose and all interference concentrations (except ascorbic acid) were 0.2 mM in phosphate buffer. Ascorbic acid was added four times: twice 0.02 mM and twice 0.002 mM. Interferences were added as follows: citric acid, sucrose, maltose, fructose, mannose, galactose, lactose, ascorbic acid (0.02 mM), ascorbic acid (0.002 mM).

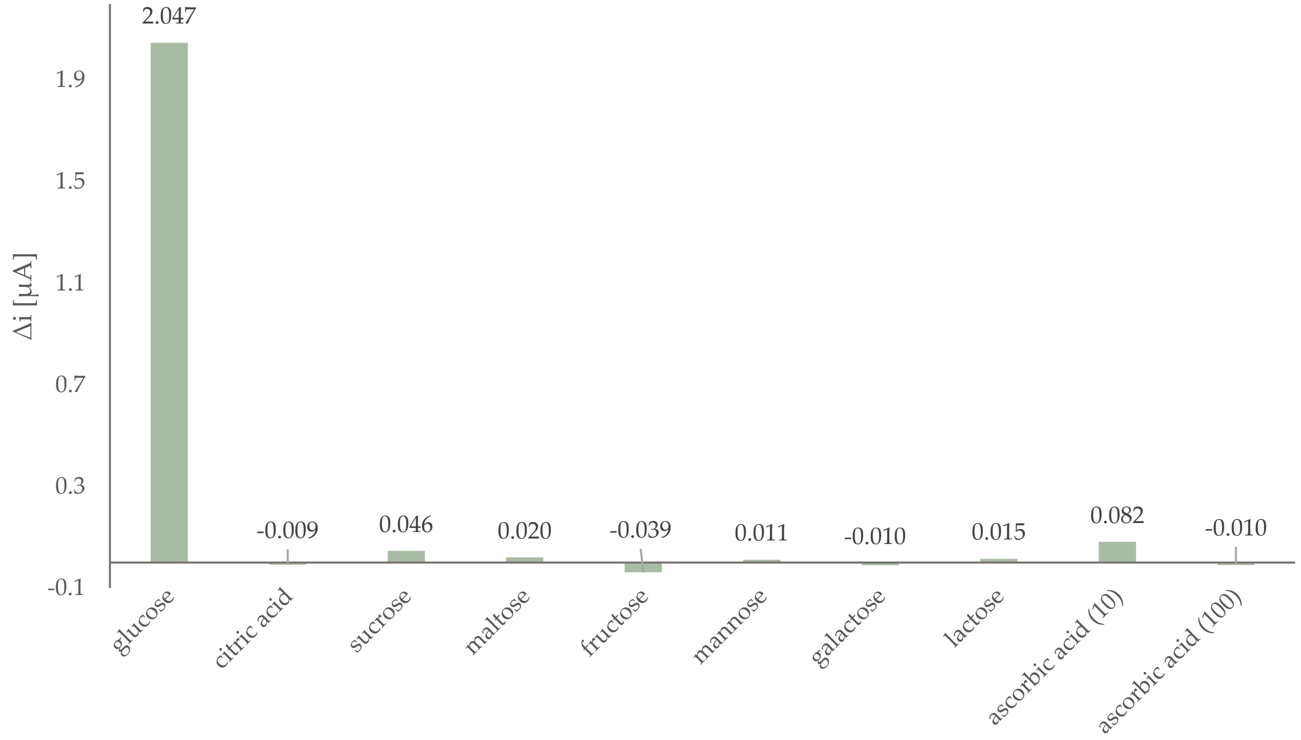

Figure S6: A visual representation of sensor current responses for chosen interferences. Amperogram for the same measurement shown in Figure S3.

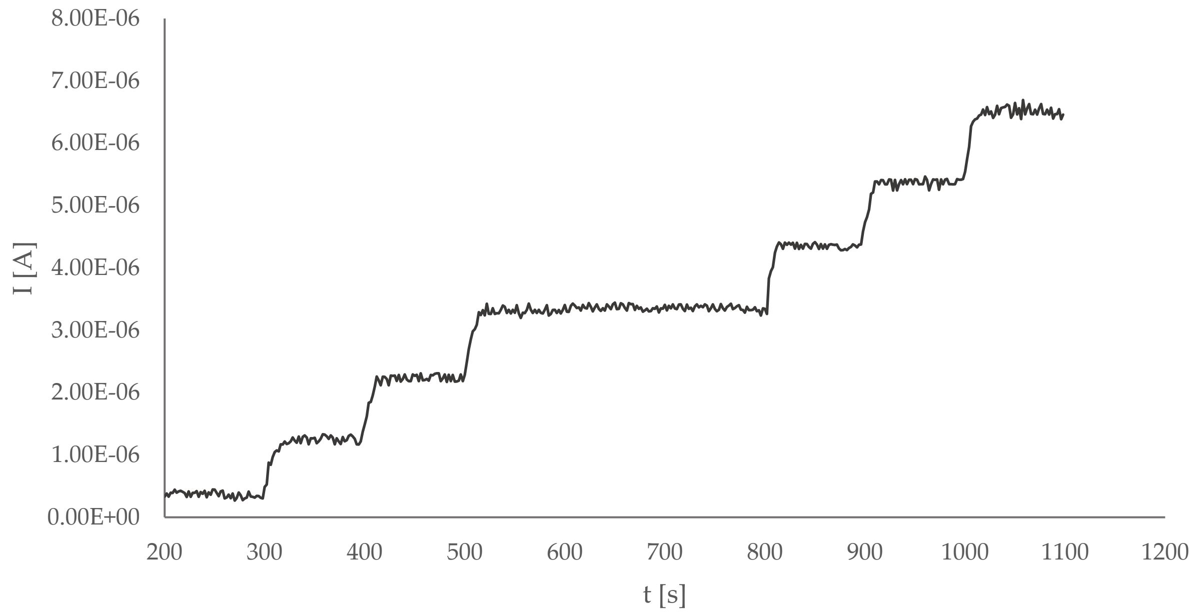

Figure S7: Amperogram for one of the real matrix measurements. The measurement procedure was following: (1) standard glucose solution was added in the electrochemical cell (at 300, 400, 500 s); (2) beer was added in the electrochemical cell (at 600, 700, 800 s); (3) spiked beer samples were added in the electrochemical cell (at 800, 900, 1000 s). The beer was spiked so that the final concentration of glucose in the electrochemical cell after every addition would change for 0.1 mM. Recovery values for glucose determination were calculated. Recovery value for this amperogram was 103.5%.
